# Efficacy and Safety of Selective Laser Trabeculoplasty among Ethiopian Glaucoma Patients

**DOI:** 10.1155/2020/7620706

**Published:** 2020-09-16

**Authors:** Jibat Gemida Soboka, Abeba T. Giorgis, Abiye M. Alemu, W. G. Hodge, Karim F. Damji

**Affiliations:** ^1^Department of Ophthalmology, College of Medical and Health Science, Haramaya University, Harar, Ethiopia; ^2^Department of Ophthalmology, School of Medicine, College of Health Sciences, Addis Ababa University, Addis Ababa, Ethiopia; ^3^Ivey Eye Institute, Schulich School of Medicine & Dentistry, University of Western Ontario, London, Ontario, Canada; ^4^Department of Ophthalmology and Visual Sciences, University of Alberta, Edmonton, Canada

## Abstract

**Background:**

Selective laser trabeculoplasty (SLT) is a safe and effective treatment modality for lowering intraocular pressure (IOP).

**Purpose:**

To determine the efficacy and safety of SLT among Ethiopian patients with primary open-angle glaucoma (POAG), pseudoexfoliation glaucoma (PXG), and ocular hypertension (OHT).

**Method:**

A prospective, nonrandomized interventional study was conducted at Menelik II Hospital, Ethiopia. Patients on antiglaucoma medication with uncontrolled IOP and those patients treated for the first time with 360 degrees of SLT were included. Success was defined as an IOP lowering of > 20% from baseline without repeat treatment.

**Result:**

A total of 95 eyes of 61 patients with a diagnosis of OAG and OHT were enrolled. The diagnosis was POAG in 55 (57.9%) eyes, PXG in 22 (23.2%) eyes, and OHT in 18 (18.9%) eyes. Seventy (73.7%) eyes were on medications, and 25 (26.3%) eyes were treated with laser as primary therapy. The mean (SD) baseline IOP and medication were 24.3 ± 2.5 mmHg and 1.29 ± 1.01, respectively. The one-year mean (SD) IOP reduction was 6.7 ± 4.2 mmHg and medication reduction was 0.26 ± 1.34. The overall IOP reduction at 12 months was 27.6%, and the success rate was 60%. The mean IOP (SD) reduction for patients who were treated for the first time with laser and on antiglaucoma medication was 6.5 ± 3.1 mmHg and 6.8 ± 2.8 mmHg, respectively. Post-SLT, patients experienced transient ocular pain, brow ache, headache, and/or blurring of vision in 31.6%, anterior chamber reaction in 36.8%, and IOP spike ≥ 6 mmHg in 11.6%.

**Conclusion:**

SLT is an effective and safe treatment modality for OHT, POAG, and PXG among Ethiopian patients either as a first-line treatment or as an adjunct to topical glaucoma treatment.

## 1. Background

The glaucomas are a diverse group of disorders that have in common an intraocular pressure (IOP) sensitive optic neuropathy, which leads to progressive visual field loss.

The aim of glaucoma therapy is to lower IOP in order to slow or arrest glaucoma progression. Currently, this goal is pursued with one or more of medication, laser treatment, or surgery. Each treatment option, however, has potential challenges with efficacy, safety, compliance, and cost.

Medical therapy can lead to local and systemic side effects and a high percentage of patients have poor adherence (68%) [1].

Laser trabeculoplasty (LTP) has the potential to decrease IOP in patients with or at risk for open-angle glaucoma (OAG) without systemic side effects and also to minimize concerns about compliance with drop therapy (depending on whether adjunctive medication is needed to achieve the target IOP range) [2, 3].

Laser trabeculoplasty (LTP) can be performed with a variety of lasers and reduces IOP by improving the facility of outflow.

Argon laser trabeculoplasty (ALT) was the first LTP procedure in the 1970s [4]. It was subsequently utilized as an adjunct to topical and oral medications or as initial treatment [5]. Although successful in lowering IOP, ALT has several side effects, most notably elevated IOP (short- and long-term spikes) and inflammation. It also coagulates the trabecular meshwork (TM) tissue, resulting in peripheral anterior synechiae, and repeat treatment has been shown to be ineffective [5].

Selective laser trabeculoplasty (SLT) was developed in 1995 by Latina and Park as an alternative to ALT [6]. SLT has a very short pulse duration (3 ns), which is shorter than the thermal relaxation time of melanin, allowing for selective photo thermolysis. Because SLT selectively targets the pigmented TM cells and has an energy level ˂ 1% of ALT, it is a gentle laser than ALT with no or minimal histologic scarring or coagulative damage to the TM, thus reducing collateral damage to surrounding tissues [6, 7].

Over the last two decades, SLT has been shown to be a safe and effective treatment modality for lowering IOP in patients with or at risk for developing OAG [2]. The preservation of the trabecular meshwork architecture and the demonstrated efficacy in lowering IOP in a variety of open-angle glaucomas make SLT a reasonable and safe alternative to argon laser trabeculoplasty. In addition, SLT has been shown to be potentially repeatable in patients who have failed previous SLT as well as previous ALT [8, 9]. Furthermore, SLT has been utilized as a primary treatment option in a variety of OAG patients including those who cannot tolerate or are noncompliant with their glaucoma medications; it also does not appear to cause TM or conjunctival damage and thus should not interfere with future microinvasive angle or external filtration surgeries. Due to its nondestructive properties and low complication rates, SLT has the potential to evolve as an ideal first-line treatment in open-angle glaucoma [4, 8].

The purpose of this study was to compare the pattern of IOP reduction and side effects following SLT in treated OHT and OAG patients.

## 2. Method and Patients

This study was a prospective, nonrandomized, interventional study conducted at Menelik II Hospital, a tertiary eye center located in the capital city, Addis Ababa, where ophthalmic patients are referred from different parts of the city and regions of the country between April 2017 and March 2018. All glaucoma patients who fulfilled the inclusion criteria were included in the study. The study was approved by the Ophthalmology Department research and publication committee of the College of Health Sciences, Addis Ababa University.

### 2.1. Eligibility Criteria

#### 2.1.1. Inclusion Criteria

  Age 40 years and older
  Early to moderate primary open-angle glaucoma (POAG) or pseudoexfoliation glaucoma (PXG)
  Phakic and pseudophakic
  At least Shaffer grade 3 open angle by gonioscopy
  OHT with risk factors for glaucoma progression (age above 40, high initial IOP, increased vertical cup/disc ratio, and thin central corneal thickness)
  IOP between 21 and 30 mmHg measured on at least two previous visits (prior to SLT) with or without medical therapy

#### 2.1.2. Exclusion Criteria

  Congenital, juvenile, inflammatory, or neovascular glaucoma
  Corneal edema or other corneal pathology precluding accurate tonometry and/or visualization of the AC angle structures
  Advanced glaucoma with vertical cup disc ratio (VCDR) > 0.85 and/or visual field defect involving the central 10 degrees
  Cataract requiring surgery

All consecutive glaucoma patients who presented to the glaucoma clinic during the study period and who fulfilled the eligibility criteria were included in the study. The purpose of the study was explained to each patient, written consent was obtained, and patient confidentiality was maintained throughout the study. Sociodemographic and clinical data, such as age, sex, history of systemic disease, and medication, antiglaucoma drugs (numbers), and duration of application of antiglaucoma drops were obtained from all patients included in the study.

Baseline IOP was taken using the Goldmann applanation tonometer. The average of 3 measurements was taken before SLT decision on different follow-up visits. Central corneal thickness was measured using a digital pachymeter (DGH 55 Technology INC, Exton, PA, USA). Gonioscopy examination was performed using Sussman four-mirror gonio lens to determine the extent of angle opening and the level of angle pigmentation. The Spaeth grading system was used for AC angle pigmentation. Dilated fundus examination under SLM using 90D lens was used to assess the retina and optic nerve head.

### 2.2. Procedure

The procedure was carried out by two senior ophthalmologists at the glaucoma clinic (ATG and AM). The initial and post-SLT questionnaires were taken by the principal investigator.
Prior to laser
    Brimonidine 0.2% single drop was applied one hour before SLT to help prevent/blunt the risk of post-SLT IOP spike.
    Pilocarpine 2% single drop was used to keep the pupil constricted and prevent peripheral-iris crowding 30 min before SLT.
    Topical anesthesia (tetracaine 0.5%) was applied 1 to 2 minutes before the procedure.
The laser procedure involved the Lumenis Selecta SLT Laser (Lumenis Inc., Santa Clara, CA), and a Latina SLT Gonio Lens (Ocular Instruments, WD, USA). To determine the optimum energy level, the laser was initially set at 0.8 mJ, and then the energy level increased or decreased by 0.1 mJ until the threshold energy for fine cavitation bubble (champagne bubbles) formation was observed. This was the energy that was utilized for subsequent spots. Treatment was delivered in single non-overlapping pulse mode placing 100 contiguous spots along 360° of the TM.
Immediately after the completion of the procedure, brimonidine 0.2% drop was applied and IOP was measured after an hour.
All patients were prescribed topical NSAIDs (diclofenac sodium 0.1%, or flurbiprofen sodium 0.03%) on QID basis for 7 days after laser treatment.

### 2.3. Data Analysis

Data included range, mean ± standard deviation, frequencies (number of cases), relative frequencies (percentages), and intereye correlation calculated using Huber regression as appropriate. A probability value (*p* value) less than 0.05 was considered statistically significant. All statistical calculations were performed with SPSS (Statistical Package for the Social Science; SPSS Inc., Chicago, IL) version 23.

### 2.4. Operational Definition

  Success was defined when IOP reduction of >20% was achieved from the baseline without repeat SLT.
  Intraocular pressure spike was defined as a transient IOP increase (vs. baseline) of at least 6 mm Hg 1 hour post-SLT.
  Ocular hypertension: one or more of the following: IOP > 21 mm Hg; suspicious disc or VCDR asymmetry of >0.2; suspicious HVF 24-2 (or similar) VF defect.
  Early glaucoma: early glaucomatous disc features (e.g., VCDR < 0.65) and/or mild VF defect not within 10° of fixation (e.g., MD better than −6 dB on HVF 24-2).
  Moderate glaucoma: moderate glaucomatous disc features (e.g., vertical VCDR 0.7–0.85) and/or moderate VF defect not within 10° of fixation (e.g., MD from −6 to −12 dB on HVF 24-2).
  Advanced glaucoma: advanced glaucomatous disc features (e.g., VCDR > 0.9) and/or VF defect within 10° of fixation (e.g., MD worse than −12 dB on HVF 24-2).

## 3. Result

A total of 95 eyes of 61 African patients were involved in the study. The mean age of the study participants was 57.3 ± 10.2 years (range, 40 to 87 years) and 49 (80.3%) were males. The glaucoma diagnosis was POAG in 55 (57.9%) eyes and PXG in 22 (23.2%) eyes. Eighteen (18.9%) eyes were diagnosed to have OHT, which are summarized in Table 1. Diabetes mellitus and systemic hypertension were noted in 21 (34.4%) and 15 (24.9%), respectively. Thirty-one (32.6%) eyes belonged to DM patients. Seventy (73.7%) eyes were on medications, and 25 (26.3%) eyes were treated with laser as primary therapy. Majority of eyes (79 (83.2%)) completed the 12-month follow-up visit.

Pre-SLT baseline IOP was 24.3 ± 2.5 mmHg (range, 21 to 30 mmHg), and pre-SLT mean number of antiglaucoma drugs used was 1.29 ± 1.01. The mean (SD) CCT and VCD were 538.8 ± 32.7 *μ*m and 0.51 ± 0.19, respectively. The mean (SD) of the total energy used was 89.82 ± 29.64 mJ (range, 39 to 188 mJ).

Overall, the mean (SD) IOP after SLT was 23.3 ± 5.1 mmHg, 19.7 ± 4.8 mmHg, 18.5 ± 4.3 mmHg, 18.8 ± 3.9 mmHg, 18.0 ± 3.3 mmHg, and 17.6 ± 3.4 mmHg at 1 hour post-SLT, 1^st^ week, 1^st^ month, 3^rd^ month, 6^th^ month, and 12^th^ month, respectively.

The mean IOP reduction and percentages of IOP at each visit were 1 mmHg (4.1%), 4.6 mmHg (18.9%), 5.8 mmHg (23.9%), 5.5 mmHg (22.6%), 6.3 mmHg (25.9%), and 6.7 mmHg (27.6%) at 1 hour post-SLT, 1^st^ week, 1^st^ month, 3^rd^ month, 6^th^ month, and 12^th^ month, respectively. At the last visit, the overall percentage of IOP reduction from the baseline was 27.6%, and those who achieved the 20% IOP reduction success were 57 (60%) eyes of which 44 (77.2%) were on medication and 13 (22.8%) were treated with laser as primary therapy. There was no statistical correlation between success and previous antiglaucoma medication (*p*=0.148).

In males, the baseline IOP was 24.6 ± 2.6 mmHg and the mean IOP reduction was 7.0 ± 3.5 mmHg (28.5%). The success rate in males was 69.8%. In females, the baseline IOP was 23.2 ± 1.7 mmHg and the mean IOP reduction was 5.3 mmHg (22.8%). The success rate in females was 62.5%. Between males and females IOP reduction, there was no statistically significant difference (*p*=0.779).

The inter-eye correlation was calculated for IOP reduction at different visits. The baseline IOP was 24.3 mmHg. Post-SLT IOP at different visits was at 1 hour 23.3 mmHg (*p* = 0.08), at 1 week 19.7 mmHg (*p* = 0.00001), at 1 month 18.7 mmHg (*p* = 0.00001), at 3 months 18.8 mmHg (*p* = 0.00001), at 6 months 18.0 mmHg (*p* = 0.00001), and at 12 months 17.7 mmHg (*p* = 0.00001).

**Figure 1:**
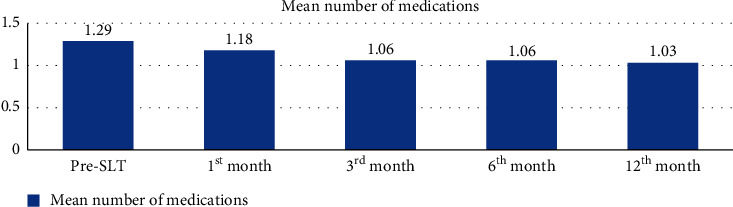
The mean number of medications used before and after SLT, Menelik II Hospital, 2018.

The mean (SD) antiglaucoma medications were at 1^st^ month 1.18 ± 0.94, 3^rd^ month 1.06 ± 0.84, 6^th^ month 1.06 ± 0.80, and 12^th^ month 1.03 ± 0.70 (Figure 1). The overall IOP and medication reduction achieved at the last visit were 6.7 ± 4.2 mmHg and 0.26 ± 1.34, respectively. From those on medications before SLT, 4.3% of them were off medication at the last visit. There was a significant correlation between pre-SLT and 12^th^ month post-SLT number of antiglaucoma medications with *p* < 0.001. The number of drugs reduced from an average of 1.3 to an average of 1.0 was statistically significant with the inter-eye correlation *p* = 0.02.

**Figure 2:**
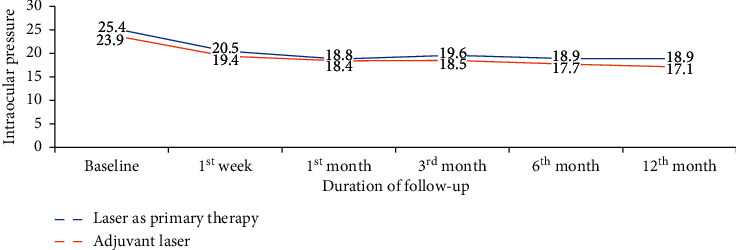
Mean IOP after SLT for laser treatment as primary therapy and adjuvant laser treatment group, Menelik II Hospital, 2018.

Those patients who were treated with laser as primary therapy with a baseline IOP of 25.4 mmHg ±2.9 mmHg had IOP reduction of 6.5 ± 3.1 mmHg at the last visit, and those who were on antiglaucoma medication with baseline IOP 23.9 ± 2.2 mmHg had IOP reduction of 6.8 ± 2.8 mmHg. There was no significant correlation between being on antiglaucoma medication and laser as primary therapy on the final visit IOP reduction (*p*=0.528).  Figure 2 shows IOP reduction between the two groups throughout follow-up visits.

**Figure 3:**
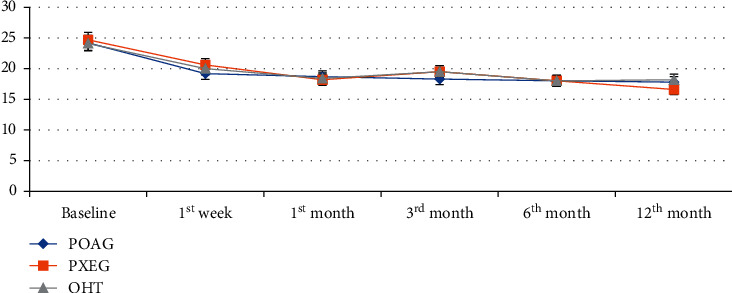
Mean IOP before and after SLT for POAG, PXG, and OHT at different follow-up visits, Menelik II Hospital, 2018.

IOP reduction at the last visit between POAG, PXG, and OHT was 6.4 mmHg, 8.1 mmHg, and 5.9 mmHg, respectively, which is shown in Table 2.  Figure 3 shows the mean IOP throughout follow-up visits among different types of glaucoma.

On the basis of success (20% IOP reduction), the success rate did not show a statistically significant difference between POAG, PXG, and OHT (*p*=0.663). From the total 57 (60%) eyes achieved success, 33 (57.1%) eyes were of POAG patients, 16 (28.1%) eyes were of PXG patients, and 8 (14%) eyes were of OHT patients.

The success achieved in each type of glaucoma was 60% in POAG, 72.7% in PEXG, and 44.4% in OHT. The highest reduction was seen in PXEG, but there was no significant correlation between the type of glaucoma and last visit IOP reduction (*p*=0.941).

The mean (SD) energy per spot and total energy used were 0.79 ± 0.23 mJ (range, 0.4 to 1.5 mJ) and 89.82 ± 29.65 mJ (range, 39 to 188 mJ), respectively. There was no significant correlation between energy used and last visit IOP reduction (*p*=0.650).

There were 31 (32.6%) eyes in the DM patients' group with baseline mean (SD) IOP of 23.9 ± 1.7 mmHg (range, 21 to 27 mmHg). Twenty-one (67.7%) eyes were on antiglaucoma medications and 10 (32.3%) eyes were treated with laser as primary therapy. The overall IOP reduction was 5.5 ± 3.6 mmHg (23.0%) and the 20% IOP reduction success was 73.9%. There was no statistically significant difference in IOP reduction between DM and non-DM patients (*p*=0.528).

There was a significant correlation between the age of the patient and 20% IOP reduction success at 12^th^ month follow-up with *p*=0.002. We did not find a significant correlation between 12^th^ month 20% IOP reduction success and the use of antiglaucoma medication before SLT (*p*=0.110). There was no significant correlation between angle pigmentation and 12-month IOP reduction (*p*=0.457). Similar to many other studies, we found greater IOP reduction (9.7 mm Hg) at the last visit in those patients with baseline IOP higher than 26 mmHg. This showed the higher pre-SLT IOP with more IOP drop after SLT which was statistically significant with *p*=0.0001. In those with IOP below 25 mmHg, the final IOP reduction was 5.7 mmHg.

One hour after SLT 31.6% of patients had ocular pain, brow ache, headache, and/or blurring of vision. On slit-lamp examination, 35 (36.8%) of eyes had AC reaction (graded from 1 to 4) of +1 in 23 (24.2%), +2 in 10 (10.2%), and +3 in 2 (2.1%); 6 (6.3%) eyes had conjunctival injection. There was no corneal edema or hyphema observed during the study period. Peripheral anterior synechiae was not observed at 6^th^ and 12^th^ months follow-up visit. IOP spikes ≥ 6 mmHg were only seen in 11 (11.6%) eyes 1 hour after SLT and in 1 (1.1%) eye after 1 week.

## 4. Discussion

In our study, we evaluated the efficacy and safety of SLT in patients with OHT as well as various types of OAG. Our patients were treated with laser as primary therapy or adjunct laser with medication. The overall IOP reduction was 27.6%, and the success rate was 60% at 1 year. This finding is comparable with a study conducted in Egypt by Ahmed et al. which noted 70% success at 18 months and with that of St. Lucia by Realini which noted 65.0% success at 1–5 years [10, 11].

IOP reduction was similar in POAG and PXG and also not different in OHT patients. This is similar to other studies.

Overall IOP reduction at different follow-up visit ranges from 4.5% to 27.6% with the highest IOP reduction noted at 12^th^ month follow-up period. Our finding is comparable with the study done by Latina et al. which demonstrated a 30% reduction of IOP at 26 weeks [4].

Similar to many other studies, we found greater IOP reduction at the last visit in those patients with baseline IOP higher than 26 mmHg which was 9.7 mmHg. In those with IOP below 25 mmHg, the final IOP reduction was 5.7 mmHg. This finding was also reported by Latina et al. which was 10.6 mmHg for those patients with baseline IOP more than 26 mmHg. However, there was no correlation between baseline IOP and success at 12^th^ month.

In our study, we found no correlation between angle pigmentation and final IOP reduction which is similar to what others have noted, e.g., Koucheki and Hashemi in Iran [10]. There was no correlation between having diabetes mellitus and IOP reduction which showed 23.0% IOP reduction and 73.9% success rate. Our finding was different from a study conducted in Iran by Koucheki which showed less effectiveness of SLT in DM patients. This may be because 67.7% of our patients were on medication whereas those in the Kouchecki study were not [10].

NSAIDs were used for post-SLT inflammation control for seven days. There was a report of 31.6% complaint after SLT and 36.8% of AC reaction. Different studies found different results for post-SLT pain and AC reaction depending on energy used, extent of treated angle, and type of glaucoma. Latina et al., Koucheki et al., and Nagar et al. found the rate of ocular discomfort in 15%, 23.5%, and 39%, respectively, and our study is in this range [10–12]. We found the rate of IOP spike to be 11.6% which is lower than what is found by Latina et al. which was 24%. This difference is likely related to pre- and post-SLT application of brimonidine in our patients.

We found a significant correlation between the number of antiglaucoma medication reduction from the baseline and the number of medication reduction at the final visit which was 0.26 ± 1.34. This finding was also observed in Ahmed et al.'s study in Egypt [13, 14].

In conclusion, in our study, we found 27.6% overall IOP reduction and 60% success rate at the last visit, and there was no lasting complication observed. The effect of SLT is similar in POAG, PXG, and OHT.

### 4.1. Recommendation

Selective laser trabeculoplasty can be used safely among Ethiopian patients with OHT and OAG as a first-line treatment or adjunct to medical management to lower the intraocular pressure and reduce the number of ocular hypotensive drugs.

## Figures and Tables

**Table 1:** Demographic and clinical characteristics of patients underwent selective laser trabeculoplasty (SLT) at Menelik II Hospital Glaucoma Clinic, 2018.

Characteristics	Value
Age	
Mean (SD)	57.3 (±10.2)

Gender, *n* (%)	
M	49 (80.3)
F	12 (19.7)

Diagnosis, *n* (%)	
POAG	55 (57.9)
PXG	22 (23.2)
OHT	18 (18.9)

**Table 2:** Mean baseline IOP in mmHg and mean IOP reduction in mmHg at the follow-up visit in POAG, PXG, and OHT, Menelik II Hospital, 2018.

	POAG (*n* = 55)	PXG (*n* = 22)	OHT (*n* = 18)
Baseline IOP	24.2	24.7	24.1
1^st^ week	5	4.1	4.1
1^st^ month	5.5	6.5	5.7
3^rd^ month	5.9	5.2	4.6
6^th^ month	6.2	6.7	6.1
12^th^ month	6.4	8.1	5.9

## Data Availability

The data used to support the findings of the study are available from the corresponding author via jibatgemida@gmail.com.

## Additional Points

There remains an unmet need for a safe, efficient, and cost-effective therapy for glaucoma patients in the developing world. Selective laser trabeculoplasty (SLT) brings a “drop-free window” of several years, removes concerns about compliance, and reduces the need for multiple drops even years later. It is also repeatable. This study is the first published use of SLT in Ethiopia. We have used SLT in our hospital over the last four years and felt that objective assessment of its efficacy and safety was crucial to help guide future use. Our study demonstrated a significant reduction in IOP (27.6%) as well as the number of medications, both of which were statistically significant. SLT is a rapid, safe, and minimally invasive procedure. The equipment is portable, and the incremental cost of treatment is small once the equipment and expertise are on-site. SLT has the potential to be a safe and effective mobile glaucoma laser treatment in our population.
